# Impact of Theoretical Comprehension on Periodontal Instrumentation Skills Development

**DOI:** 10.1002/jdd.13921

**Published:** 2025-04-27

**Authors:** Se‐Lim Oh, Deborah Jones, Sheryl Syme, Oksana Mishler, Hanae Saito

**Affiliations:** ^1^ Department of Advanced Oral Sciences and Therapeutics University of Maryland School of Dentistry Baltimore Maryland USA

**Keywords:** clinical skill, periodontics, predoctoral periodontal education, theoretical comprehension

## Abstract

**Objectives:**

The study aimed to investigate the impact of theoretical comprehension in periodontics on clinical instrumentation skills development through a longitudinal assessment.

**Methods:**

Classes of 2023 (*n*1 = 126) and 2024 (*n*2 = 128) were included. Both classes took the same case‐based examination (CBE) in the second year and similar CBEs in the third and fourth years. The two classes undertook the same instrumentation skill tests during the second and fourth years. Two‐way repeated measures ANOVA, correlation tests, and linear regression analyses (LRA) were conducted with a significance level of *α* = 0.05.

**Results:**

The class of 2024 exhibited significantly lower performances on all three CBEs (*p* = 0.0002) than the class of 2023. Nonetheless, instrumentation skills development between the two classes did not differ based on the scores in the skill tests (*p* = 0.53), with identical clinical experience reflected as clinic points. A positive correlation between the clinic points and the fourth‐year scaling competency examination scores and no correlation between the clinic points and the fourth‐year CBE scores were observed. There were no correlations between the fourth‐year CBE and fourth‐year scaling competency examination scores based on *R* ^2^ values of 0.01 for the class of 2023 and 0.06 for the class of 2024 derived from LRA.

**Conclusion:**

The outcomes of the didactic assessments were not predictive of performance in periodontal instrumentation. The lack of correlation between didactic and skill test scores suggests that cognitive understanding and manual skills are distinct domains, each requiring targeted instructional strategies and assessment methods.

## 1 Introduction

Predoctoral periodontal education encompasses a substantial body of theory derived from preclinical and clinical research [1]. Various educational approaches, primarily didactic lectures [2], are employed to help dental students attain comprehensive theoretical understanding in periodontics. Case‐based and problem‐based learning are often integrated with didactic lectures to build students’ competence in assessment, diagnosis, prevention, treatment planning, and nonsurgical management of periodontal diseases [2].

Students must also acquire basic clinical skills, including nonsurgical periodontal therapy, which involves understanding the rationale behind periodontal instrumentation. Nonsurgical periodontal instrumentation is performed to reduce the bacterial load and suppress gingival inflammation [3]. Effective periodontal instrumentation requires skillful use of periodontal instruments to remove deposits without damaging tissues. Therefore, teaching periodontal instrumentation via simulation‐based learning (SBL) and clinical practice is an essential part of predoctoral periodontics courses [4].

Predoctoral periodontics courses administer various assessment methods to gauge students’ levels of periodontal theoretical comprehension and instrumentation skills. The most common method to measure theoretical comprehension levels is written examinations, primarily using multiple‐choice questions (MCQs) [2, 5]. Well‐constructed MCQs objectively evaluate students’ factual recall of lecture contents [5] but may not fully evaluate their conceptual understanding and critical thinking skills [6, 7]. In contrast, case‐based open‐ended questions present standardized cases along with uniform questions [8], simulating real‐life clinical scenarios [7]. These questions require students to apply their knowledge from previous and current curricula, clinical experiences, and critical thinking skills. Therefore, case‐based examinations (CBEs) more effectively assess students’ in‐depth comprehension and problem‐solving abilities than MCQs [7].

Technical skill assessments are performed during preclinical and clinical education. Students’ performances on typodonts in preclinical skill assessments and on patients in clinical skill assessments are evaluated by calibrated faculty members in controlled settings [9]. Studies have reported no correlations between outcomes in preclinical practical grades and clinical performances on patients [10, 11]. Nevertheless, preclinical assessments using typodonts may reflect a wide range of students’ readiness for clinical patient care, which may provide a valuable milestone in skill assessments [12].

Evaluating how theoretical understanding translates into clinical skills development is essential for improving healthcare educational programs [13, 14]. However, the relationship between theoretical understanding and clinical skills development in predoctoral periodontics is underexplored. This observational study investigated the impact of theoretical comprehension in periodontics on clinical instrumentation skills development through longitudinal assessment of two cohorts, classes of 2023 and 2024. The correlations between the fourth‐year CBE and patient‐based scaling competency examination scores were assessed. The null hypothesis was that the CBE scores would have no effect on the scaling competency examination scores.

## 2 Materials and Methods

This observational study was conducted under a non‐human subject research (NHSR) protocol approved by the institutional review board (IRB) at the University of Maryland, Baltimore (HP‐00096962), as this study was conducted retrospectively, and there was no direct interaction with the participants.

### 2.1 CBEs With Open‐Ended Questions

This study used student performances in CBEs as indicators of their theoretical comprehension. Table 1 summarizes the contents of the CBEs from the second to fourth years. The same course directors for each course graded the open‐ended questions for the classes of 2023 and 2024. The rubric for these questions is detailed in a previous publication (Table S1) [15].

**TABLE 1:** Periodontics courses and case‐based examinations during predoctoral dental education.

Year	Lecture contents	Examination format	Contents for case‐based examinations
2nd year	Periodontitis and nonsurgical treatment approaches	Three short‐answer and six open‐ended questions	Diagnosis with classification, risk factors, local contributing factors, and prognosis
3rd year	Surgical treatment approaches	Eight open‐ended and three multiple‐choice questions	Assessment, diagnosis, and treatment planning toward the periodontal initial therapy
4th year	No lecture	Eight open‐ended and four multiple‐choice questions	Diagnosis, initial therapy, reevaluation of the initial therapy, formulation of the surgical therapy, and maintenance

A remote learning module was implemented for the class of 2023 during their second year due to the COVID‐19 pandemic shutdown; an onsite learning module was implemented for the class of 2024 in the second year [15]. Otherwise, both classes underwent onsite learning modules during the third and fourth years. The same clinical case and set of questions were presented to the classes of 2023 and 2024 at the second‐year CBEs [15]. This ensured that both classes faced identical scenarios and questions, allowing for a consistent assessment between the two classes. During their third and fourth years, different cases of comparable complexity were presented to the two classes, but each class received the same case in their third‐ and fourth‐year CBEs. Similar questions were included in the examinations for both classes. This repetition was intended to assess the progress in the student's understanding and management of the case over time.

### 2.2 Clinical Periodontal Education

At the University of Maryland School of Dentistry, students must complete a minimum of three periodontal patient cases to graduate. Completion of a periodontal case indicates that a student performed a periodontal treatment plan presentation, any required periodontal instrumentations, disease control procedures, and reevaluation of the periodontal therapy. Clinic points, which are based on whether the procedure is incomplete or complete, are awarded to each periodontal procedure performed and completed under the supervision of calibrated periodontal faculty.

In preparation for the periodontal treatment plan presentation, students must perform full periodontal charting and complete a comprehensive computer‐based form, which includes clinical and radiographic findings, diagnoses, prognoses, and treatment plans. All findings are presented to a periodontal faculty member at the periodontal treatment planning appointment, where the faculty provides feedback on the student's presentations.

### 2.3 Periodontal Instrumentation Skill Assessments

The second‐year practical examination, concluding the preclinical education, is a typodont‐based instrumentation skill examination; its evaluation focuses on identifying the correct working end, proper adaptation of periodontal instruments, and effective instrumentation stroke. The fourth‐year scaling competency examination, marking the end of clinical education, is a patient‐based instrumentation skill examination; its evaluation focuses on calculus detection, calculus removal, and soft tissue management.

A remote SBL module was implemented for the class of 2023 [16], while an onsite SBL module was employed for the class of 2024 during their second year. There was no difference in clinical education between the two classes. Calibrations for dental hygiene faculty members have been conducted since 2016, along with the development and refinement of the evaluating rubrics [17]. Once statistical consistency was achieved [17], the hygiene faculty members discussed the performances of the first 10 students each year to ensure ongoing consistency. The same calibrated faculty members evaluated student performances in both the second‐year practical and fourth‐year scaling competency examinations for the two classes. The same grading rubrics were used (Tables S2 and S3) [11, 18].

### 2.4 Statistical Analysis

A post hoc power analysis showed that a sample size of 126 exhibited a power of 0.94 to detect the medium effect size (*f* ^2 ^= 0.1) with one predictor [19]. Descriptive statistics were prepared for all variables. Student performances in CBEs and periodontal instrumentation skill assessments were compared between the classes and within the classes over time using two‐way repeated measures Analysis of Variance (ANOVA) followed by Tukey multiple comparison tests. Pearson correlation coefficient tests were conducted to examine the relationships between clinic points and two outcomes—the fourth‐year CBE and scaling competency examination scores. A simple linear regression model was used to evaluate the correlation between students' performances in the fourth‐year CBE and the scaling competency examination for each class. Data analysis was performed with a software program (GraphPad Prism version 10.2.3; GraphPad Software, Inc.). The level of significance was set at *α* = 0.05.

## 3 Results

A total of 254 students from the two cohorts (the class of 2023; *n*1 = 126 and the class of 2024; *n*2 = 128) were included in this study. The two classes had comparable numbers of female (65 vs. 61) and male (69 vs. 59) students.

Table 2 summarizes class performances in CBEs and periodontal instrumentation skill assessments. Both classes showed their highest performances at the third‐year CBE among didactic assessments. Both classes showed significantly improved performances in instrumentation skill assessment from the second year to the fourth year. Regarding clinical training, both classes had almost identical clinical training experiences under the supervision of periodontal faculty members based on clinical points and the number of periodontal cases.

**TABLE 2:** Summary of class performances in case‐based examinations and periodontal instrumentation skill assessments. % scores with mean ± standard deviation in examinations.

Domain		Class of 2023 (*n*1 = 126)	Class of 2024 (*n*2 = 128)
Case‐based examinations			
2nd‐year case‐based examination		78 ± 10.5	69 ± 17
3rd‐year case‐based competency examination		87 ± 7	84 ± 7
4th‐year case‐based competency examination		82 ± 6	78 ± 7
Instrumentation skill assessments			
2nd‐year practical examination		85 ± 17.5	86 ± 17
Clinical training	Clinic points	171 ± 54.1	172 ± 47.2
	Number of cases	6 ± 1	6 ± 1
4th‐year scaling competency examination		90 ± 8.6	89 ± 7.9

**FIGURE 1:**
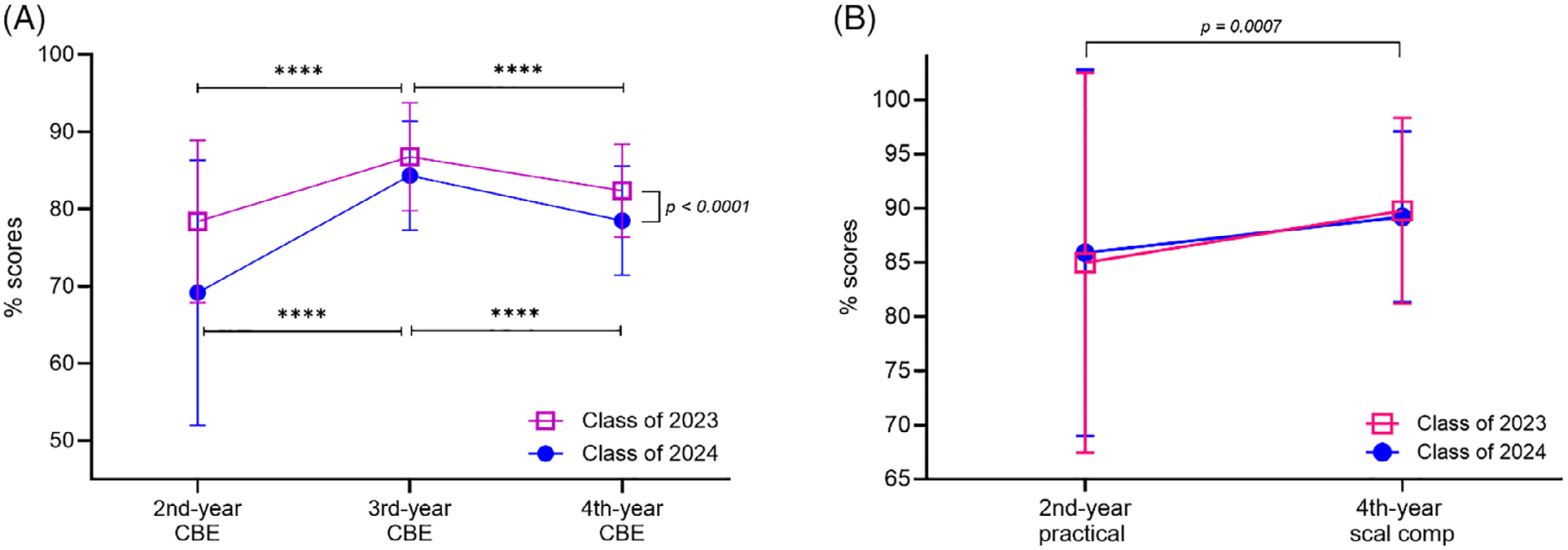
Comparisons between the classes of 2023 and 2024. (A) Two‐way repeated measures ANOVA for comparison of the case‐based examination (CBE) scores between the two classes. Tukey multiple comparisons in the CBE scores within the classes, ****p < 0.0001. (B) Two‐way repeated measures ANOVA for comparison of instrumentation skill tests between the two classes.

Figure 1 presents the results of comparisons between the classes and within the classes over time in CBEs and skill assessments. The class of 2024 showed significantly lower performances in all CBEs across all time periods when compared to the class of 2023 (Figure 1A; two‐way repeated measures ANOVA, class effect, *p *< 0.0001). Significant variations were observed over time in both classes, showing the lowest performance in the second‐year CBE and the highest performance in the third‐year CBE (multiple comparison tests, *p* < 0.0001). The two classes did not show differences at each skill test (Figure 1B; two‐way repeated measures ANOVA, interaction between class and time, *p* = 0.53). The scores in skill assessments were significantly improved from the second year to the fourth year in both classes (two‐way repeated measures ANOVA, time, *p* = 0.0007).

Table 3 presents the correct answer rates in assigning a stage and a grade for the periodontitis cases in each examination in the two classes. The performances of the class of 2023 were significantly superior in most questions to the class of 2024, except for the periodontitis grade assignment on the third‐year CBE.

**TABLE 3:** Comparisons of the correct answer rates in assigning a stage and a grade for the cases between the two classes. CBE, case‐based examination. The number of correct answer/the number of incorrect answer (the correct answer rate).

		Class of 2023 (*n*1 = 126)	Class of 2024 (*n*2 = 128)	*p* value^a^
2nd‐year CBE	Stage	117/9 (93%)	95/33 (74%)	< 0.0001
	Grade	91/35 (72%)	71/57 (55%)	0.006
3rd‐year CBE	Stage	124/2 (98%)	119/9 (93%)	0.03
	Grade	114/12 (90%)	107/21 (84%)	0.1
4th‐year CBE	Stage	122/4 (97%)	110/18 (86%)	< 0.0001
	Grade	101/25 (80%)	81/47 (63%)	0.003

^a^ Chi‐square test

Table 4 presents the results of Pearson correlation coefficient tests examining the association between the clinic points and the four‐year CBE and scaling competency examination scores (*N* = 254). While there was a positive correlation between the clinic points and the fourth‐year scaling competency examination scores (*p* = 0.0007), no correlation was observed between the clinic points and the fourth‐year CBE scores (*p* = 0.1).

**TABLE 4:** Correlations between the clinic points and the fourth‐year case‐based examination (CBE) and scaling competency (scal comp) examination scores.

	The fourth‐year CBE (80.4 ± 6.8)	The fourth‐year scal comp (89.5 ± 8.2)
Clinic points (171.7 ± 50.6)	*r* = 0.1, 95% CI [−0.01–0.2], *p* = 0.1	*r* = 0.21^***^, 95% CI [0.1–0.32], *p* = 0.0007

*Note: N* = 254. Mean ± standard deviation. *r *= Pearson correlation coefficient.

Abbreviation: CI, confidence interval.

*** *p* < 0.001.

**FIGURE 2:**
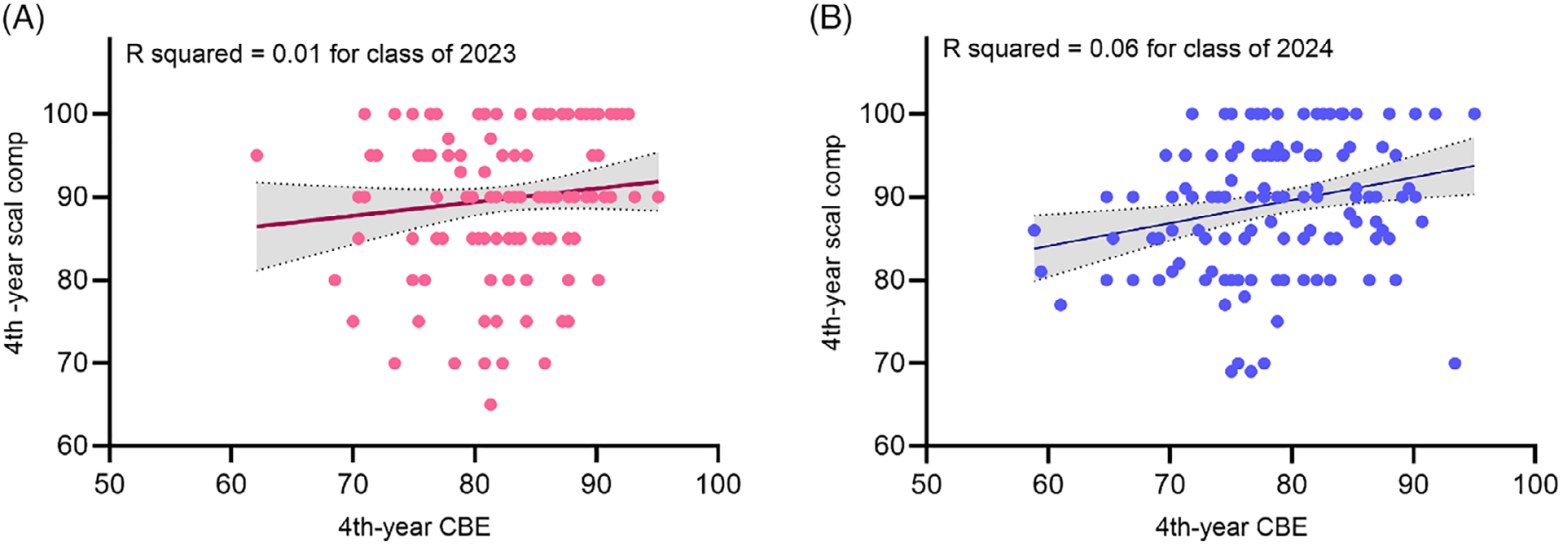
Simple linear regression between the fourth‐year case‐based examination (CBE) and scaling competency (scal comp) examination for the class of 2023 (A) and the class of 2024 (B).

Figure 2 presents plots from simple linear regression analysis for each class to evaluate the correlation between the CBE and scaling competency examination scores. In the class of 2023, there was no correlation between the two examination scores (Figure 2A; *F* = 1.67, *p* = 0.198, *R* ^2 ^= 0.01). In the class of 2024, although the relationship between the two scores was statistically significant (Figure 2B; *F* = 8.17, *p* = 0.005*, R* ^2 ^= 0.06), only 6% of the variation in the scaling competency examination scores could be explained by this model. Therefore, the observed relationship was not practically meaningful.

## 4 Discussion

The study found no correlation between the didactic and clinical skills assessments and accepted the null hypothesis; although a comprehensive knowledge of periodontal concepts guides the development of periodontal treatment plans, it had no effect on psychomotor skill development in periodontal instrumentation.

The class of 2023 consistently outperformed the class of 2024 in all CBEs. Despite the assumption that onsite learning is more effective, a previous study found no difference in learning effectiveness between remote and onsite modules [15]. The lower performance of the class of 2024 is unlikely due to instructional methods. Other factors might have contributed to this difference. The class of 2024 may have had lower engagement levels [20]. Class dynamics, including peer support and collaboration, might have been more favorable for the class of 2023 [21]. Additionally, the class of 2023's remote learning experience might have enhanced their technological skills and adaptability, giving them an edge in computer‐based assessments [22].

Students in both classes showed improved ability to retrieve and apply second‐year course content during their third‐year assessments. Third‐year lectures presented new materials on surgical treatments, while the third‐year CBE covered nonsurgical periodontal therapy, reflecting the second‐year content. Correct answers on periodontitis staging and grading increased from the second to third year (Table 3), suggesting students applied both prior knowledge and clinical experience. Therefore, clinical experience might improve their theoretical understanding.

While clinical skills rely on sound theoretical knowledge, clinical performance should be assessed by direct observation in real clinical settings [23]. The fourth‐year periodontics course implemented a patient‐based scaling competency examination. However, administering this test is highly demanding, as it requires many resources, including time, spaces, calibrated faculty, and patients with similar conditions [11]. To address the aforementioned challenges, studies have investigated the relationship between theoretical knowledge and clinical skill tests, as written examinations are less extensive [11].

However, many studies found weak or no correlation between written examination scores and clinical skill test results in medical education [24, 25, 26]. Mixed results were reported in dental education. While a high correlation between knowledge‐based and clinical examinations was reported in first‐year student cohorts [27], no correlations were observed between didactic and practical test scores from the third to fifth years [28].

Knowledge acquisition for clinical skills is often measured by written examinations with MCQs at the low levels of Miller's pyramid model [23]. CBEs with open‐ended questions require higher critical thinking skills, not relying on memorizing the facts [7]. Therefore, assessments for theoretical comprehension and clinical skills cannot replace each other and should be implemented separately.

Almost identical performances were observed in the fourth‐year patient‐based scaling competency examinations among four consecutive fourth‐year dental classes with similar clinical experiences [11]. If students have clinical experience with an average of six periodontal cases, it is expected that they attain the appropriate instrumentation skills. To alleviate the effort involved with the patient‐based examination, it is proposed to implement a standardized typodont‐based examination to evaluate and confirm students’ clinical skills in effective calculus removal as the fourth‐year instrumentation skill test.

The study results also show that student performances on written examinations were considerably lower than in skill assessments in periodontics (Table 2). To improve theoretical comprehension and align it with clinical training, we suggest integrated and reflective practice. Practical examinations and clinical training should include components where students provide the rationale behind their procedures and anticipated outcomes from periodontal procedures.

The generalizability of the study findings is limited due to a few factors. First, although the sample size was large, the study population was limited to two cohorts from a single institution. Second, student‐level factors related to the periodontics courses were not measured and, therefore, could not be considered in this study, although these factors may influence students’ performances, especially on knowledge assessments. To capture student‐level factors, future studies should employ a mixed‐method approach that combines subjective and objective measures. Survey questionnaires, especially those with open‐ended questions, can be administered to assess subjective aspects such as student motivation, study habits, engagement, and prior and current knowledge. Learning analytics from course management systems can be utilized to track objective student learning behaviors.

## 5 Conclusion

The outcome of didactic assessments in periodontics was not predictive of performance in periodontal instrumentation. Although didactic knowledge is essential for developing a comprehensive periodontal treatment plan, it had no effect on psychomotor skill development. This suggests that cognitive understanding and manual skills are distinct domains, each requiring specific instructional strategies and test methods. To achieve optimal periodontal clinical outcomes, targeted instructional strategies to enhance comprehensive knowledge, aligning with clinical skills, should be implemented.

## Author Contributions

S.‐L.O contributed to the contention or design of the work, the acquisition, statistical analysis, interpretation of data for the work, drafting the work or revising it critically for important intellectual content, final approval of the version to be published, and agreement to be accountable for all aspects of the work. D.J. contributed to teaching in the course and the clinic, drafting the work or revising it critically for important intellectual content, final approval of the version to be published, and agreement to be accountable for all aspects of the work. S.S. contributed to teaching in the course and the clinic, drafting the work or revising it critically for important intellectual content, final approval of the version to be published, and agreement to be accountable for all aspects of the work. O.M. contributed to teaching in the course and the clinic, drafting the work or revising it critically for important intellectual content, final approval of the version to be published, and agreement to be accountable for all aspects of the work. H.S. contributed to teaching in the course and the clinic, drafting the work or revising it critically for important intellectual content, final approval of the version to be published, and agreement to be accountable for all aspects of the work.

## Conflicts of Interest

The authors declare no conflicts of interest.

## Supporting information



Supporting Information
