# Supplementary material for: Impact of Theoretical Comprehension on Periodontal Instrumentation Skills Development
Source: J Dent Educ. 2025 Apr 27;90(1):99–105. doi: 10.1002/jdd.13921 (PMC12800723; doi:10.1002/jdd.13921)
Supplement: Supplementary file 1 — Supporting Information [file JDD-90-99-s001.docx]

**Supplementary Table 1**. The scoring rubric for open-ended questions.

| Score | Criteria |
| --- | --- |
| 4 | Correct answer with listing all key supporting evidence, using proper terms |
| 3 | Correct answer with missing key supporting evidence, using proper terms |
| 2 | Correct answer with mixing correct and incorrect supporting evidence, using proper terms |
| 1 | Correct answer with listing only incorrect supporting evidence, not using proper terms |
| 0 | Incorrect answer to the question |

**Supplementary Table 2**. The rubric for the four-year scaling competency examination.

| **Calculus Detection (20 Points)** |
| --- |
| 1. Independent and correct calculus site selection by student (20) 2. 2-3 sites need faculty intervention and re-selected (10) 3. 4 or more sites need faculty intervention and re-selected (-31) * |
| **Sub- and Supra-gingival Calculus/Stain/Plaque/Removal (60 Points)** |
| 1. No calculus at selected sites detected, Plaque-free and relatively stain free (60) 2. Calculus detected at ≤2 sites (50) 3. Calculus detected at ≤5 sites (40) 4. Calculus detected at more than >5 sites. Significant plaque and/or stain remains at more than 2 sites (-31) * |
| **Soft tissue management (20 Points)** |
| 1. No tissue trauma evident, no extraneous tissue tags, hemostasis observed/no coagulum remnants (20) 2. Minor tissue trauma evident (15) 3. Major tear in tissue/multiple minor sites, significant Tissue tags/granulation left (-31)* |
| **Others** |
| 1. Major issue in pain control (-31)* 2. Asepsis violation (-31)* |

*Critical errors are automatic failures.

**Supplementary Table 3**. The modified rubric for the second-year periodontal practical examination.

| **Gracey 1/2 (20 points)** | |
| --- | --- |
| **Critical Failure**   - Incorrect instrument used (-20) - Incorrect working end used (-20) - Using the same working end both on the mesial and distal surfaces (-20) - Scaling “backwards” (-20) - Not performed on the palatal side (-10) | - Inappropriate pen grasp (-2) - No/inappropriate fulcrum (-2) - Not inserting at the middle (-2) |
|  | - Overall stroke was too weak/ little pull (-2) - Little/no overlapping stroke/not continuous (-2) - Not going interproximally (-2) - Not enough rolling of the tip of the instrument (-2) - Instrument adaptation is too open or too closed (-2) |
|  | - Other comments |
| **Gracey 11/12 (20 points)** | |
| **Critical Failure**   - Incorrect instrument used (-20) - Incorrect working end used (-20) - Using the instrument on the distal surface of the tooth (-20) - Scaling “backwards” (-20) - Not performed on the palatal side (-10) | - Inappropriate pen grasp (-2) - No/inappropriate fulcrum (-2) - Incorrect insertion point (-2) |
|  | - Overall stroke was too weak/ little pull (-2) - Little/no overlapping stroke/not continuous (-2) - Not going interproximally (-2) - Not enough rolling of the tip of the instrument (-2) - Instrument adaptation is too open or too closed (-2) |
|  | - Other comments |
| **Gracey 13/14 (20 points)** | |
| **Critical Failure**   - Incorrect instrument used (-20) - Incorrect working end used (-20) - Using the instrument on the mesial surface of the tooth (-20) - Scaling “backwards” (-20) - Not performed on the palatal side (-10) | - Inappropriate pen grasp (-2) - No/inappropriate fulcrum (-2) - Incorrect insertion point (-2) |
|  | - Overall stroke was too weak/ little pull (-2) - Little/no overlapping stroke/not continuous (-2) - Not going interproximally (-2) - Not enough rolling of the tip of the instrument (-2) - Instrument adaptation is too open or too closed (-2) |
|  | - Other comments |
| **Universal Columbia 13/14 (20 points)** | |
| **Critical Failure**   - Incorrect instrument used (-20) - Incorrect working end used (-20) - Flipping the instrument on the same tooth surface (-20) - Scaling only the half surface on the tooth (-20) - Scaling “backwards” (-20) - Not performed on the palatal side (-10) | - Inappropriate pen grasp (-2) - No/inappropriate fulcrum (-2) - Incorrect insertion point (-2) |
|  | - Overall stroke was too weak/ little pull (-2) - Little/no overlapping stroke/not continuous (-2) - Not going interproximally (-2) - Not enough rolling of the tip of the instrument (-2) - Instrument adaptation is too open or too closed (-2) |
|  | - Other comments |
